# Large-Scale Functional Genomics Screen to Identify Modulators of Human β-Cell Insulin Secretion

**DOI:** 10.3390/biomedicines10010103

**Published:** 2022-01-04

**Authors:** Iwona Szczerbinska, Annamaria Tessitore, Lena Kristina Hansson, Asmita Agrawal, Alejandro Ragel Lopez, Marianne Helenius, Andrzej R. Malinowski, Barak Gilboa, Maxwell A. Ruby, Ramneek Gupta, Carina Ämmälä

**Affiliations:** 1 Department of Discovery Biology and Pharmacology, Novo Nordisk Research Centre Oxford, Oxford OX3 7FZ, UK; armalinowski@ymail.com (A.R.M.); mwry@novonordisk.com (M.A.R.); caaq@novonordisk.com (C.Ä.); 2 Department of Discovery Technology and Genomics, Novo Nordisk Research Centre Oxford, Oxford OX3 7FZ, UK; azte@novonordisk.com (A.T.); vawl@novonordisk.com (A.A.); azrl@novonordisk.com (A.R.L.); bkgi@novonordisk.com (B.G.); 3 Department of Computational Biology, Novo Nordisk Research Centre Oxford, Oxford OX3 7FZ, UK; lena@hpldesign.se (L.K.H.); nqmh@novonordisk.com (M.H.); rmgp@novonordisk.com (R.G.); 4 Department of Health Technology, Technical University of Denmark, 2800 Copenhagen, Denmark

**Keywords:** siRNA screen, T2D, β-cell, EndoC-βH1, EndoC-βH5, text mining, glucose-stimulated insulin secretion, GSIS, large-scale

## Abstract

Type 2 diabetes (T2D) is a chronic metabolic disorder affecting almost half a billion people worldwide. Impaired function of pancreatic β-cells is both a hallmark of T2D and an underlying factor in the pathophysiology of the disease. Understanding the cellular mechanisms regulating appropriate insulin secretion has been of long-standing interest in the scientific and clinical communities. To identify novel genes regulating insulin secretion we developed a robust arrayed siRNA screen measuring basal, glucose-stimulated, and augmented insulin secretion by EndoC-βH1 cells, a human β-cell line, in a 384-well plate format. We screened 521 candidate genes selected by text mining for relevance to T2D biology and identified 23 positive and 68 negative regulators of insulin secretion. Among these, we validated ghrelin receptor (*GHSR*), and two genes implicated in endoplasmic reticulum stress, *ATF4* and *HSPA5*. Thus, we have demonstrated the feasibility of using EndoC-βH1 cells for large-scale siRNA screening to identify candidate genes regulating β-cell insulin secretion as potential novel drug targets. Furthermore, this screening format can be adapted to other disease-relevant functional endpoints to enable large-scale screening for targets regulating cellular mechanisms contributing to the progressive loss of functional β-cell mass occurring in T2D.

## 1. Introduction

Type 2 diabetes (T2D) is a chronic metabolic disorder affecting almost half a billion people worldwide [1]. It is a leading cause of death due to its co-morbidities such as kidney failure, heart attack, or stroke [2]. β-cell insufficiency, the failure of β-cells to meet increased demand for insulin caused by insulin resistance, leads to the development of overt T2D. It has been estimated that at the time of T2D diagnosis only about 50% of functional β-cell mass remains [3,4]. The regulation of glucose-stimulated insulin secretion (GSIS) is complex and the underlying molecular mechanisms are still not fully understood. Identifying novel regulators of insulin secretion could lead to the development of new therapeutic interventions for T2D.

Most studies investigating cellular mechanisms regulating insulin secretion utilize non-human models such as mouse or rat β-cells [5,6,7]. However, molecular and functional differences between rodents and human β-cells argue for the use of human-derived in vitro models [8,9] for the discovery of novel therapeutic targets. The generation of EndoC-βH1 cells, the first and to date only glucose-responsive human-derived β-cell line, enabled the development of human-relevant in vitro assays [10]. As an equivalent to fasted and fed states in vivo, EndoC-βH1 cells secrete basal insulin under low glucose and increase secretion when stimulated with high glucose, respectively. Secretion potentiators such as IBMX can provide additional information regarding mechanisms further augmenting glucose-stimulated secretion (e.g., cAMP-mediated pathways).

Phenotypic screening is a proven strategy for the discovery of first-in-class medicines [11]. Efforts to identify targets for phenotypic screening have benefitted greatly from the increased availability of diverse data types and computational power. For example, as we have previously described [12], data-driven text mining can score proteins according to relevance to T2D-related biology and identify lists of putative targets at a greater scale than the capacity of current screening approaches. As EndoC-βH1 phenotypic screening efforts so far have been limited to a semi-high-throughput 96-well assay format [13], there is a need to develop robust, miniaturized phenotypic assays for large-scale screening to enable efficient target discovery and realize the full potential of combined in silico/in vitro target discovery approaches.

Here we established and applied a 384-well format human-centric screen to functionally interrogate 521 genes coding for putative novel T2D drug targets identified through in silico semantic text mining. Specifically, we examined the effects of siRNA-mediated loss-of-function of the candidate genes on basal, glucose-stimulated, and IBMX augmented glucose-stimulated insulin secretion in EndoC-βH1 cells. We generated over 12,000 data points across 21 plates and identified a total of 91 genes acting as positive, or negative, modulators of insulin secretion. Finally, we validated selected hits in independent experiments and extended findings to the recently developed EndoC-βH5 cells.

## 2. Materials and Methods

### 2.1. Text Mining

The approach used to identify proteins related to T2D was based on a previously described text mining method [12]. Here, we expanded the biology underpinning T2D by increasing the list of proteins that are either known or postulated to be involved in disease prevention or progression, either directly or through interactions with other known proteins. By including more than 300 proteins as seed to define biological concepts of interest for a semantic text mining of PubMed abstracts, more than 500 proteins were identified as being either directly or indirectly linked to T2D related biology. UniProt IDs of text mining genes are provided in Table S1.

### 2.2. EndoC-βH1 Cell Culture

For routine culture, EndoC-βH1 cells were propagated in OPTIβ1 (Human Cell design, OB1-100). Cells were cultured on TPP plastic (Helena Biosciences, 93100T, Gateshead, UK) precoated with 1% ECM gel (Sigma, E1270, Gillingham, UK), 1% Penicillin/Streptomycin (Gibco, 15140122), and 2 µg/mL Fibronectin (Sigma, F1141) in High Glucose DMEM (Gibco, 41965-039) 1 h prior to seeding. Cells were subcultured using Trypsin (Sigma, T3924-100ml) every 7–10 days at a seeding density of 70,000–75,000 cells/cm^2^. Trypsin was neutralized in 20% FBS (Heat inactivated; Gibco, 10500-064)/PBS (Gibco, 10010-023).

### 2.3. EndoC-βH5 Cell Culture

EndoC-βH5 cells (Human Cell Design) were thawed and cultured following the manufacturer’s protocol. Cells were seeded 20,000 cells/well into 384-well plates (Perkin Elmer CellCarrier-384 Ultra, 6057300, Seer Green, UK). Reverse transfection was performed as described in Section 2.6. Media was refreshed 4 days post-seeding, GSIS was performed on day 7 as described in Section 2.4.

### 2.4. Glucose Stimulated Insulin Secretion (GSIS)

24 h prior to assay, cells were incubated in starvation media (Human Cell Design). The assay was performed on Hamilton VANTAGE System Liquid Handler. On the day of assay, cells were washed twice with 0.1% BSA (Fraction V, fatty acid free, Roche, 10775835001) in βKrebs (Human Cell Design) and starved in BSA/ βKrebs (no glucose) for 1 h at 37 °C in 5% CO_2_. Insulin secretion assay was performed in 50 µL (384-well format) or 100 µL (96-well format) in BSA/βKrebs under the following conditions: no glucose, 20 mM glucose (Sigma, G8644-100 mL), 20 mM glucose + 50 µM IBMX (Sigma, I5879-100 mg) and 20 mM glucose +1 nM exendin-4 (Sigma, E7144) (only for EndoC- βH5) for 1 h at 37 °C. Supernatants were collected for insulin quantification.

### 2.5. Insulin Quantification

Secreted insulin was quantified using Insulin High Range HTRF kit (CisBio, 61IN1PEG, Saclay, France). Antibodies were dispensed (anti-insulin Eu3+ and XL655) using Hamilton VANTAGE Liquid Handler with a ratio of 2:1 (25 µL) on an assay plate (Perkin Elmer OptiPlate-384, 6007290). Five microliters of supernatant collected during GSIS was added to the same plate. The following day, the plate was read on a BMG ClARIOstar Plus using settings recommended by the kit’s manufacturer.

### 2.6. siRNA Transfection in 96-Well and 384-Well Format

Cells were reverse transfected at final concentration of 25 nM siRNA, and 0.1 µL (384-well plates) or 0.2 µL (96-well plates) RNAiMAX (Invitrogen, 13778-150, Loughborough, UK) in OptiMEM (Gibco, 13778-150). Then, 15,000 EndoC-βH1, 20,000 EndoC-βH5 cells/well for 384-well plate and 40,000 EndoC-βH1 cells/well for a 96-well were reverse transfected. Catalog numbers for siRNAs used for optimization and validation are listed in Table S2. siRNAs against text mining genes were cherry-picked from siRNA libraries: Human Druggable Subsets (Dharmacon, G-104675-E2, Cambridge, UK), Human Drug Targets (Dharmacon, G-104655-E2), Human Genome (Dharmacon, G-106500-E2).

### 2.7. siRNA Randomisation on the Screening Plates

siRNAs against text mining genes were picked from a library containing 17,959 siRNAs (details in Table S3) using UniProt IDs. The library information was stored in a database system from which the siRNAs were picked using Oracle through python (v3.8.4) with the package cx_Oracle (v8.0.1). The well positions on the destination plates were assigned randomly using random.sample() script from the built-in python package “random” to draw without replacement from a listed set of available well positions on each destination plate. Each destination plate contained siRNAs targeting candidate genes in triplicates (*n* = 3) with their positions randomized across the plate. Furthermore, it was ensured by sorting the source well positions, that all siRNAs from the same source plate were picked sequentially to avoid multiple freeze-thaw cycles.

### 2.8. siRNA Assay Plates Preparation

0.5 nmol of the whole genome siRNA library was received in ECHO-qualified 384PP source plates. A master library was prepared as 10 µM stocks by briefly centrifuging plates and resuspending in 50 µL of siRNA buffer (Horizon, #B-002000-UB-100, Cambridge, UK) diluted to 1X in RNase-free water (Horizon, #B-003000-WB-100). Four 7.5 µL daughter sets were prepared from the master library in ECHO-qualified 384LDV source plates. The entire set was assigned plate barcodes for automated transfer on the Labcyte Access system and TEMPO scheduler. siRNA transfer picklist was generated as described in Section 2.7 with wells reserved for control genes. Next, 100 nL siRNAs were transferred onto pre-coated 384-well destination plates (Perkin Elmer CellCarrier-384 Ultra, 6057300, Seer Green, UK) using Labcyte ECHO 650 Cherry Pick software and 384LDV_AQ_B2 calibration. The destination plates were kept in a tissue culture hood for 30 min for drying and plates were sealed and stored at −20 °C.

### 2.9. High-Throughput siRNA Screen

15,000 EndoC-βH1 cells/well were reverse transfected in 384-well plates as described in Section 2.6. Each plate contained siRNAs targeting candidate genes in triplicates (*n* = 3) with their positions randomized across the plate. In addition, control siRNAs targeting *INSULIN*, *PLK1*, *ZMIZ1*, and *HNF4A* as well as non-targeting siRNAs were included on each plate. Six days following seeding and reverse transfection, GSIS was performed under three conditions (0 mM glucose, 20 mM glucose, and 20 mM glucose + IBMX), with each condition on a separate plate replica (in total 7 sets of 3 plates). siRNA plates were prepared as described in Section 2.8. GSIS and insulin quantification was performed as described in Section 2.4 and Section 2.5, respectively. After GSIS cells were fixed using Fix/Perm solution (BD, 554714), stained with Hoechst 33342 (dilution 1:5000, Invitrogen, H3570) and imaged on a CV8000 for cell number quantification. 

### 2.10. Strictly Standardized Mean Difference SSMD

Strictly standardized mean difference SSMD method was used to quantify effect size for hit selection from the screen [14]. β was calculated as: $\beta =\frac{X_{1}-X_{2}}{\sqrt{s_{1}^{2}+s_{2}^{2}}}$. Where β is the effect size, *X* is the mean, and *s* is the standard deviation.

### 2.11. RNA Extraction and qPCR

For cells cultured in a 96-well format, RNA was extracted with Dynabeads mRNA DIRECT Purification Kit (Invitrogen, 10563755) following the manufacturer’s protocol. Volumes for each step were scaled down (cells were lysed in 90 µL and mixed with 10 µL of beads in lysis buffer, washes were performed in 100 µL and RNA was eluted in 8 µL). DNase treatment and reverse transcription were performed with SuperScript IV VILO Master Mix with ezDNase Enzyme (Invitrogen, 11766500) following the manufacturer’s protocol. Cells cultured in a 384-well format were lysed and cDNA was generated with TaqMan Gene Expression Cells-to-CT Kit (Invitrogen, 4391851C and A35377) following the manufacturer’s protocol. qPCR was performed using TaqMan Fast Advanced Master Mix (Invitrogen, 4444557) in 10 µL final reaction containing 2 µL of cDNA in 384-well plates on the Biorad CFX384 (the same protocol for cDNA obtained from cell cultured in 96-well and 384-well format). The relative abundance of transcripts was calculated using ΔΔC(T) method. TaqMan probes used in the study are listed in Table S4.

### 2.12. Statistical Analysis

Data are presented as the mean ± standard deviation (SD). GraphPad Prism 9.0.1 was used for statistical analysis. *t*-test was used for the analysis of two continuous variables and one-way ANOVA for comparison of multiple groups. non-significant (ns) *p*-value >0.05, * *p*-value < 0.05, ** *p*-value < 0.01, *** *p*-value < 0.001.

### 2.13. Viability Assay

Viability was measured by Cell Titer Glo (Promega, G7571) following the manufacturer’s protocol.

### 2.14. Western Blot

Cells were washed with cold DPBS (Gibco, 14190-094) and lysed in RIPA buffer (ThermoFisher, 89901, Horsham, UK) supplemented with protease inhibitor cocktail (ThermoFisher, 78446). Proteins were quantified using a BCA Protein Assay Kit (ThermoFisher, 23225) according to the manufacturer’s instruction. Proteins were resolved on gel (Bio-Rad, Horsham, UK, 4561094) and transferred to a membrane (Invitrogen, Waltham, MA, USA, IB24002) using iBlot 2 Dry blotting system following manufacturer’s instructions (Invitrogen, IB21001). Blots were incubated with primary antibodies against actin (1:1000; Abcam, Cambridge, UK, ab8226) and insulin (1:500; Abcam, ab181547).

## 3. Results

### 3.1. Establishment of GSIS Assay and siRNA Transfection in 384-Well Plate Format

**Figure 1:**
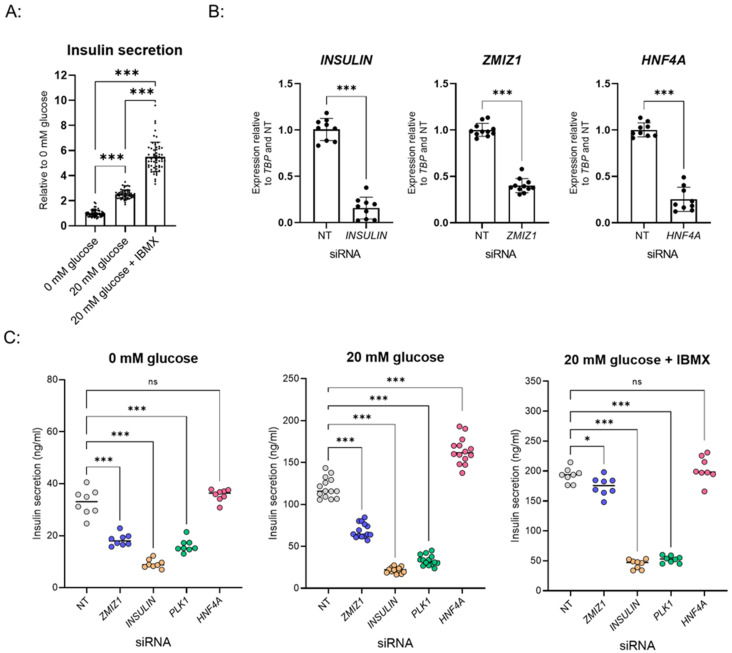
Establishment of siRNA knockdown and GSIS assay in EndoC-βH1 for a high-throughput screen in 384-well format. (**A**) Glucose-stimulated insulin secretion (GSIS) assay in EndoC-βH1 cells cultured in 384-well format. Data points are the mean ± SD of independent wells *n* = 60 for each condition. *** *p*-value < 0.001, one-way ANOVA. (**B**) Relative ex-pression of INSULIN, ZMIZ1 and HNF4A after siRNA knockdown in a 384-well format. Data points are the mean ± SD of independent wells from three experiments. *** *p*-value < 0.001, *t*-test. (**C**) Effects of control siRNAs (non-targeting [NT] control, ZMIZ1, INSULIN, PLK1, HNF4A) on GSIS in EndoC-βH1 cells in 384-well format. Data points are the mean ± SD of independent wells *n* = 8 for 0 mM glucose and 20 mM glucose + IBMX, *n* = 14 for 20 mM glucose from one experiment. ns (non-significant) *p* value > 0.05, * *p*-value < 0.05, *** *p*-value < 0.001, one-way ANOVA.

To benchmark the behavior of our EndoC-βH1 cells we performed glucose-stimulated insulin secretion (GSIS) in the previously published 96-well format (Figure S1A) [13]. In this format, high glucose (20 mM glucose) increased insulin secretion 2-fold relative to the basal condition (0 mM glucose) (Figure S1A). The addition of IBMX, a phosphodiesterase inhibitor, together with high glucose (20 mM glucose + IBMX treatment) increased insulin secretion 5-fold relative to the basal condition (Figure S1A). To increase assay throughput for screening we developed an automated protocol for EndoC-βH1 culture and the GSIS assay in a 384-well format. Therein, EndoC-βH1 responded with 2–3-fold and 5–6-fold induction of secreted insulin when stimulated with 20 mM glucose and 20 mM glucose + IBMX, respectively (Figure 1A).

To facilitate high-throughput knockdown, we developed a reverse transfection protocol in 384-well format and benchmarked it against a published 96-well procedure [13]. For initial visual validation of the knockdown protocol, we targeted polo-like kinase 1 (*PLK1*), a gene essential for EndoC-βH1 proliferation [13]. As expected, transfection with *PLK1* siRNA resulted in cell death in both 96-well and 384-well formats when compared to cells treated with non-targeting (NT) siRNA control (Figure S1B). Efficiency of knockdown was then quantified at the mRNA level for a panel of control siRNAs. Specifically, siRNAs for *INSULIN*, zinc finger MIZ-type containing 1 (*ZMIZ1*), and hepatocyte nuclear factor-4 alpha (*HNF4A*) significantly decreased their target mRNA in the 96-well (Figure S1C) and 384-well format (Figure 1B). As 6 days of siRNA treatment was sufficient to decrease protein levels of INSULIN in cell lysate (Figure S1D), we chose this time point for the screen.

To identify positive and negative controls for the screen, we assessed the effects of siRNA targeting genes expected to alter insulin secretion [13]. Knockdown of *INSULIN* or *PLK1* resulted in a significant decrease in insulin secretion across all conditions (0 mM glucose, 20 mM glucose, 20 mM glucose + IBMX) (Figure 1C). In line with previous studies, knockdown of *ZMIZ1* diminished insulin secretion, particularly in the 0 mM glucose and 20 mM glucose conditions (Figure 1C) [13,15]. Conversely, knockdown of *HNF4A* significantly increased insulin secretion in the high glucose condition (Figure 1C). Collectively, we verified siRNAs targeting *INSULIN*, *PLK1*, and *ZMIZ1* as negative controls and *HNF4A* as a positive control for inclusion in the screen.

### 3.2. High-Throughput siRNA Screen with GSIS as Readout

**Figure 2:**
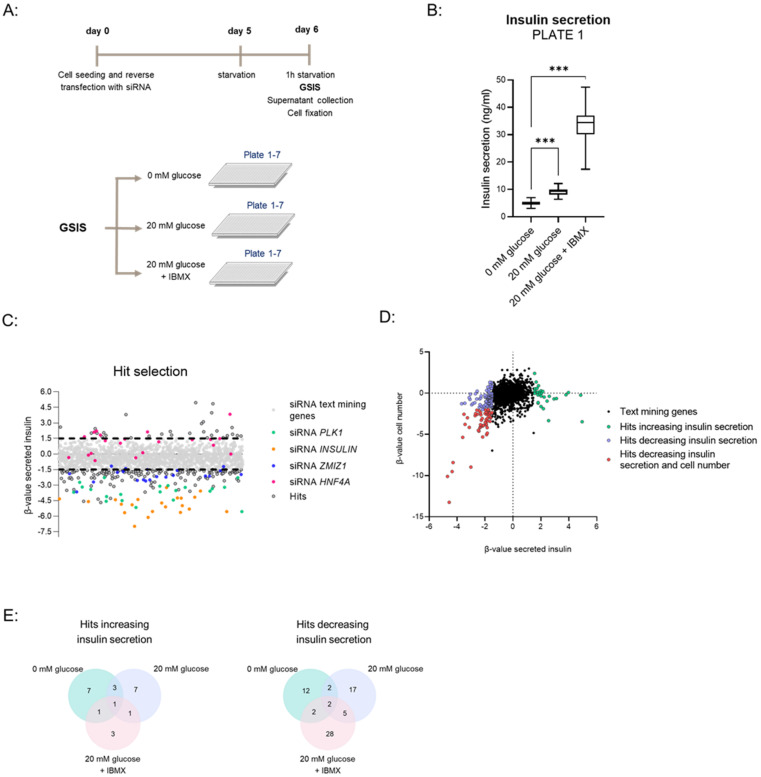
siRNA high-throughput screen in EndoC-βH1 cells with a GSIS readout. (**A**) Simplified workflow of the siRNA screen with GSIS in EndoC-βH1 cells. (**B**) Insulin secretion data for non-targeting control (siRNA NT) for a representative plate (plate 1) in the three tested conditions (0 mM glucose, 20 mM glucose, 20 mM glucose + IBMX). Box plots show min max of *n* = 33 for siRNA NT for Plate 1. *** *p*-value < 0.001, one-way ANOVA. (**C**) Dot plot showing β-values of secreted insulin following siRNA treatment across all plates and GSIS conditions. Β-values for controls are indicated by colored dots and for hits by grey dots. Black dotted lines denote cut-offs ≥1.5 and ≤−1.5 for hit selection. (**D**) Dot plot of β-value of secreted insulin (x-axis) versus β-value of cell number (y-axis) for text mining genes across all plates and GSIS conditions. Hits increasing, hits decreasing insulin secretion and hits decreasing both insulin secretion and cell number are indicated by different colors. (**E**) Venn diagrams showing numbers of hits increasing and decreasing insulin secretion identified in different GSIS conditions.

Having established the GSIS assay and siRNA transfection protocol, and selected genes to include as controls, we performed a loss-of-function siRNA screen to identify genes impacting insulin secretion. Briefly, siRNAs targeting 521 genes were distributed across seven assay plates. A simplified workflow of the screen is shown in Figure 2A, with more details in Figure S2A.

Stimulation with 20 mM glucose and 20 mM glucose + IBMX resulted in the expected induction of insulin secretion in wells treated with non-targeting siRNA on each set of plates (Figure 2B—Plate 1, Figure S2A—Plates 2–7).

The strictly standardized mean difference (SSMD) method was used to score the siRNA effect size by the difference between siRNA of interest (positive, negative controls, or text mining genes) and non-targeting control [14]. This analysis was carried out within each 384-well plate. As expected, knockdown of *INSULIN* and *PLK1* negatively affected insulin secretion consistently across all assay plates and GSIS conditions, resulting in a β-value < −2 (Figure S2C). Similarly, depletion of *ZMIZ1* diminished insulin secretion with the strongest effect in the 20 mM glucose condition (β < −2 for 20 mM glucose, Figure S2C). Conversely, knockdown of *HNF4A* increased insulin secretion after stimulation with 20 mM glucose (β-value > 1.5–2, Figure S2C). These results confirmed that the screen provides a robust platform to identify both positive and negative modulators of insulin secretion. 

We set up stringent β-value cut-offs of ≥1.5 and ≤−1.5 to identify hits either increasing or decreasing insulin secretion, respectively (Figure 2C). To exclude effects driven by cell viability, we omitted genes that decreased both insulin secretion and cell number (Figure 2D). This analysis revealed 23 and 68 genes whose depletion increased or decreased insulin secretion in at least one of the tested conditions, respectively (Figure 2E). The hits are listed in Table S5, including their UniProt IDs, gene names and β-values for insulin secretion, and cell numbers for each GSIS condition. Among the hits we identified genes whose depletion is known to alter GSIS, such as endoplasmic reticulum to nucleus signaling 1 (*ERN1*) [16], pyruvate dehydrogenase kinase 4 (*PDK4*) [17,18,19], and Serine/Threonine kinase 11 (*STK11*) [20] (Table S5), increasing confidence in our method.

### 3.3. Validation of Hits

**Figure 3:**
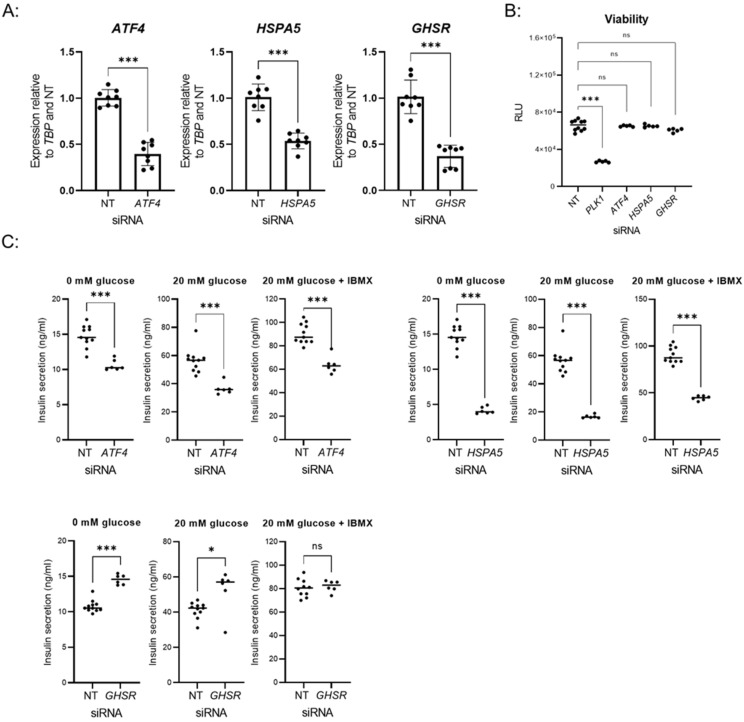
Independent validation of selected hits. (**A**) Relative expression of *ATF4*, *HSPA5*, and *GHSR* after knockdown with respective siRNAs in 384-well format. Data points show the mean ± SD of independent wells (*n* = 8) from two experiments. *** *p*-value < 0.001, *t*-test. (**B**) Viability of cells after knockdown. Data points showing relative light unit (RLU) are the mean ± SD of independent wells (*n* ≥ 5) from one experiment. ns *p* value > 0.05, *** *p*-value < 0.001, one-way ANOVA. (**C**) GSIS assay performed after transfection with following siRNAs: non-targeting (NT) control, *ATF4*, *HSPA5*, *GHSR*. Mean ± SD of *n* = 11 for siRNA NT and *n* = 6 siRNA *ATF4*, *HSPA5*, *GHSR* of independent wells from one experiment. ns *p* value > 0.05, * *p*-value < 0.05, *** *p*-value < 0.001, *t*-test.

For independent validation of the hits from the large-scale screen, we selected activating transcription factor 4 (*ATF4*) and heat-shock protein family A (Hsp70) member 5 (*HSPA5*) whose knockdown decreased insulin secretion in at least one of the conditions tested and growth hormone secretagogue receptor (*GHSR*) whose depletion augmented insulin secretion in low and high glucose concentration (0 mM glucose and 20 mM glucose) (Figure S3). To confirm that siRNA knockdown of *ATF4*, *HSPA5*, and *GHSR* was efficient, we performed qPCR on independent samples (Figure 3A). Depletion of *ATF4*, *HSPA5*, or *GHSR* did not affect cell viability as assessed by ATP content, an indicator of metabolically active cells (Figure 3B). As expected, control siRNA against *PLK1* significantly reduced cell viability (Figure 3B). Independent GSIS validation for *ATF4*, *HSPA5*, and *GHSR*, with an increased number of replicates (*n* = 6) for each condition, reproduced the screening data with depletion of *ATF4* and *HSPA5* diminishing insulin secretion and knockdown of *GHSR* enhancing insulin secretion (Figure 3C).

### 3.4. Adaptation of the Screening Setup to EndoC-βH5, a New in Vitro β-Cell Model

**Figure 4:**
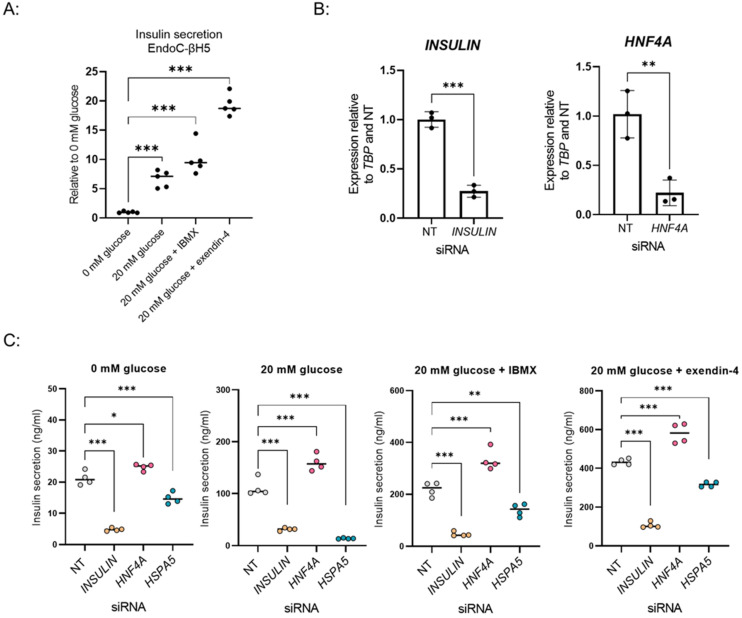
EndoC-βH5 cells can be adapted to siRNA screen. (**A**) GSIS assay in EndoC-βH5 cells cultured in 384-well format. Data points are the mean ± SD of independent wells (*n* = 5) from one experiment for 0 mM glucose, 20 mM glucose, 20 mM glucose + IBMX, and 20 mM glucose + exendin-4. *** *p*-value < 0.001, one-way ANOVA. (**B**) Relative expression of INSULIN and HNF4A after knockdown with respective siRNAs in EndoC-βH5 cells in 384-well format. Data points are the mean ± SD of independent wells (*n* = 3) from one experiment. ** *p*-value < 0.01, *** *p*-value < 0.001, *t*-test. (**C**) Knockdown effect of INSULIN, HNF4A and HSPA5 on GSIS in EndoC-βH5 cells. Data points are the mean ± SD of independent wells (*n* = 4) from one experiment for 0 mM glucose, 20 mM glucose, 20 mM glucose + IBMX and 20 mM glucose + exendin-4. * *p*-value < 0.05, ** *p*-value < 0.01, *** *p*-value < 0.001, one-way ANOVA.

Finally, we explored using our screening workflow for a recently developed human β-cell model, EndoC-βH5. These cells show an improved response to high glucose (6-fold induction of insulin secretion) and preserved response to the glucagon-like peptide 1 receptor (GLP1R) agonist exendin-4 (Figure 4A), which is absent in EndoC-βH1 (data not shown). We adapted our automated 384-well set-up for EndoC-βH5 and tested whether our established siRNA reverse transfection protocol performs robustly in this model. siRNAs targeting *INSULIN* and *HNF4A* significantly decreased their respective transcripts when compared to the NT control, indicating that EndoC-βH5 can be efficiently targeted with siRNAs (Figure 4B).

As in EndoC-βH1 cells, knockdown of *INSULIN* and *HSPA5* (a negative regulator of insulin secretion identified in the screen) decreased insulin secretion in all GSIS conditions including stimulation by exendin-4. *HNF4A* knockdown significantly increased secreted insulin in all conditions, including 0 mM glucose treatment, not affected by the knockdown in the EndoC-βH1 assay (Figure 4C, Figure 1C).

## 4. Discussion

Here, we performed the first large-scale siRNA screen to identify genes regulating insulin secretion from a set of candidate genes selected by a text mining approach. We developed a 384-well assay format with increased throughput compared to a previously reported study [13], and to facilitate interrogation of candidate genes derived from in silico approaches at scale. In order to increase the translational value of our strategy, we took advantage of the glucose-responsive β-cell line, EndoC-βH1 [10]. To enable robust identification of disease-relevant factors, we used a sensitive GSIS endpoint to determine hits in three separate functional conditions. This rich phenotypic readout has the potential to uncover targets modulating diverse aspects of insulin secretion and inform future directions for mode-of-action studies.

Our screen was able to identify positive and negative regulators of insulin secretion. Several of the genes identified in the screen have been shown to regulate insulin secretion in previous studies, increasing the confidence in our method. For instance, our finding that *STK11* knockdown results in enhanced insulin secretory activity is in line with results generated in a knockout mouse model [20]. Moreover, we identified *ERN1* as a negative regulator of insulin secretion and thereby replicated data obtained in a β-cell-specific conditional knockout study [16]. Notably, a proportion of previously proposed targets for T2D turned out as hits in a specific GSIS condition as exemplified by *PDK4*. This gene, whose depletion showed increased insulin secretion only in high glucose, is upregulated in T2D patients, and therapeutical interventions to inhibit its activity are under development [17,18,19].

Furthermore, we independently validated selected hits previously reported in non-human, or with little existing evidence linking them to insulin secretion. For example, we verified *GHSR* as a positive regulator, consistent with a study showing increased insulin secretion in *GHSR* after global and β-cell-specific knockout in mice [21,22]. *ATF4* and *HSPA5*, independently validated as negative regulators, have not been previously implicated in GSIS, but are important players in endoplasmic reticulum (ER) stress response [23,24]. Clinical and genetic evidence suggests that ER stress is one of the molecular mechanisms underlying β-cell dysfunction [25,26]. *ATF4* has been implicated in protection against dedifferentiation and β-cell loss under conditions of ER stress [23,27]. *HSPA5*, a master regulator of ER homeostasis, has been shown to have protective roles against ER stress-induced apoptosis [24,28].

Besides hits that are in line with the current literature, some genes yielded conflicting results. For example, glucokinase (*GCK*) knockdown led to an increase in insulin secretion in basal (0 mM glucose) conditions. In contrast, reduced levels of *GCK* are linked to impaired GSIS in T2D patients [29,30]. This discrepancy highlights the fact that not all findings from an in vitro model will translate to humans.

We note that our approach has several limitations. The efficiency of siRNA-mediated knockdown, which is known to vary between genes, was not quantified in our screen. CRISPR-Cas9 technology could be used to knock out genes resulting in complete loss-of-function. Recently, a pooled genome-wide CRISPR-Cas9 screen with a cellular insulin content readout has been performed in EndoC-βH1 cells [31]. While our approach presents a lower throughput compared to a genome-wide CRISPR screen, it assesses for a more complex and functional phenotype.

The text mining genes were not selected based on the direction of modulation and our loss-of-function screen may have missed genes whose gain-of-function could influence insulin secretion. As this phenotypic screen focused on identifying factors affecting insulin secretion in β-cell monocultures, it is not suitable to capture genes involved in the regulation of other aspects of β-cell function or acting through the paracrine effect on other cell types present in the islets. While our screening pipeline included cell number as an estimate of viability or proliferation, we cannot exclude the possibility that gene depletion negatively impacted cell health in other ways. Finally, due to technical challenges, we were unable to automate cell lysis in the 384-well format and could not quantify cellular insulin content.

We demonstrated that our screening procedures can be easily modified to accommodate EndoC-βH5 cells. This recently developed model more closely recapitulates properties of human β-cells in vivo including improved insulin secretion and response to GLP1R agonists, in the absence of proliferation (Human Cell Design). EndoC-βH5 could provide an alternative for GSIS-based screens once characterized in-depth and benchmarked against human islets. Furthermore, our assay format can be adapted to address additional aspects of β-cell biology. For instance, our screen can be easily modified to incorporate a disease-modeling condition, such as FGF2-induced β-cell dedifferentiation [32] or glucolipotoxicity [33], to further increase the disease relevance of our approach.

## Data Availability

The data presented in this manuscript are available from the corresponding author upon reasonable request.

## Supplementary Materials

The following supporting information can be downloaded at: https://www.mdpi.com/article/10.3390/biomedicines10010103/s1. Figure S1: Establishment of siRNA knockdown and GSIS assay in EndoC-βH1 cells; Figure S2: siRNA high-throughput screen in EndoC-βH1 cells with a GSIS readout; Figure S3: Insulin secretion (β-value) for the hits selected for validation.; Table S1. UniProt IDs of text mining genes screened in this study; Table S2. Catalog numbers of siRNAs used in this study; Table S3: Details of the Dharmacon master siRNA library; Table S4: Assay IDs of TaqMan probes used in the study; Table S5: Hits from siRNA screen.
