# Supplementary material for: Large-Scale Functional Genomics Screen to Identify Modulators of Human β-Cell Insulin Secretion
Source: Biomedicines. 2022 Jan 4;10(1):103. doi: 10.3390/biomedicines10010103 (PMC8773179; doi:10.3390/biomedicines10010103)
Supplement: Supplementary file 1 [file biomedicines-10-00103-s001.zip › biomedicines-1508980-supplementary.pdf]

biomedicines

MDPI

Supplementary Materials

Large-Scale Functional Genomics Screen to Identify Modulators of Human β-Cell Insulin Secretion

Iwona Szczerbinska [1,*], Annamaria Tessitore [2], Lena Kristina Hansson [3], Asmita Agrawal [2], Alejandro Ragel Lopez [2], Marianne Helenius [3,4], Andrzej R. Malinowski [1], Barak Gilboa [2], Maxwell A. Ruby [1], Ramneek Gupta [3] and Carina Ämmälä [1]

1. Department of Discovery Biology and Pharmacology, Novo Nordisk Research Centre Oxford, Oxford, UK; armalinowski@ymail.com (A.R.M.), mwry@novonordisk.com (M.A.R.), caaq@novonordisk.com (C.Ä.)
2. Department of Discovery Technology and Genomics, Novo Nordisk Research Centre Oxford, Oxford, UK; azte@novonordisk.com (A.T.), vawl@novonordisk.com (A.A.), azrl@novonordisk.com (A.R.L.), bkgi@novonordisk.com (B.G.)
3. Department of Computational Biology, Novo Nordisk Research Centre Oxford, Oxford, UK; lena@hpldesign.se (L.K.H.), nqmh@novonordisk.com (M.H.), rmgp@novonordisk.com (R.G.)
4. Department of Health Technology, Technical University of Denmark, DK-2800 Kongens Lyngby, Denmark

* Correspondence: iwsz@novonordisk.com

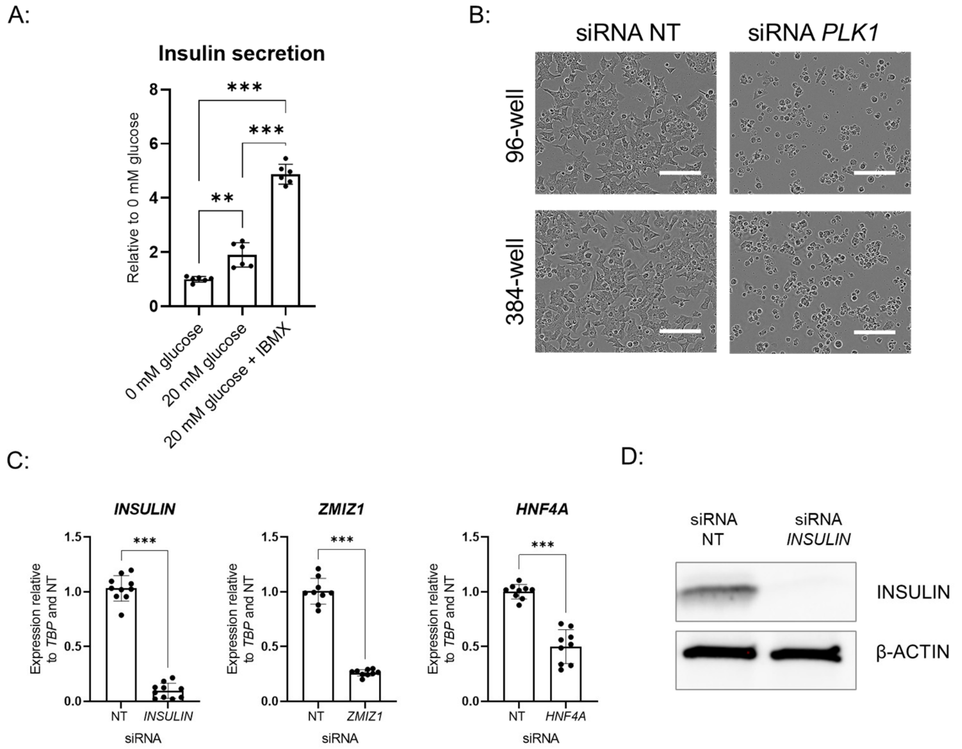

Figure S1. Establishment of siRNA knockdown and GSIS assay in EndoC-βH1 cells. **(A)** Glucose stimulated insulin secretion (GSIS) assay in EndoC-βH1 cells cultured in a 96-well format. Data points are the mean ± SD n = 6 for each condition. $^{**}p$ value <0.01, $^{***}p$ value <0.001, one-way ANOVA. **(B)** Representative images of cells after *PLK1* and non-targeting control (NT) siRNA knockdown in EndoC-βH1 cells cultured in 96-well and 384-well format. Scale bar 100 μm. **(C)** Relative expression of *INSULIN*, *ZMIZ1* and *HNF4A* after siRNA knockdown in a 96-well format. Data points are the mean ± SD n = 9 of independent wells from three experiments. $^{***}p$ value <0.001, *t*-test. **(D)** Western blot analysis of INSULIN and β-ACTIN after NT and *INSULIN* siRNA knockdown.

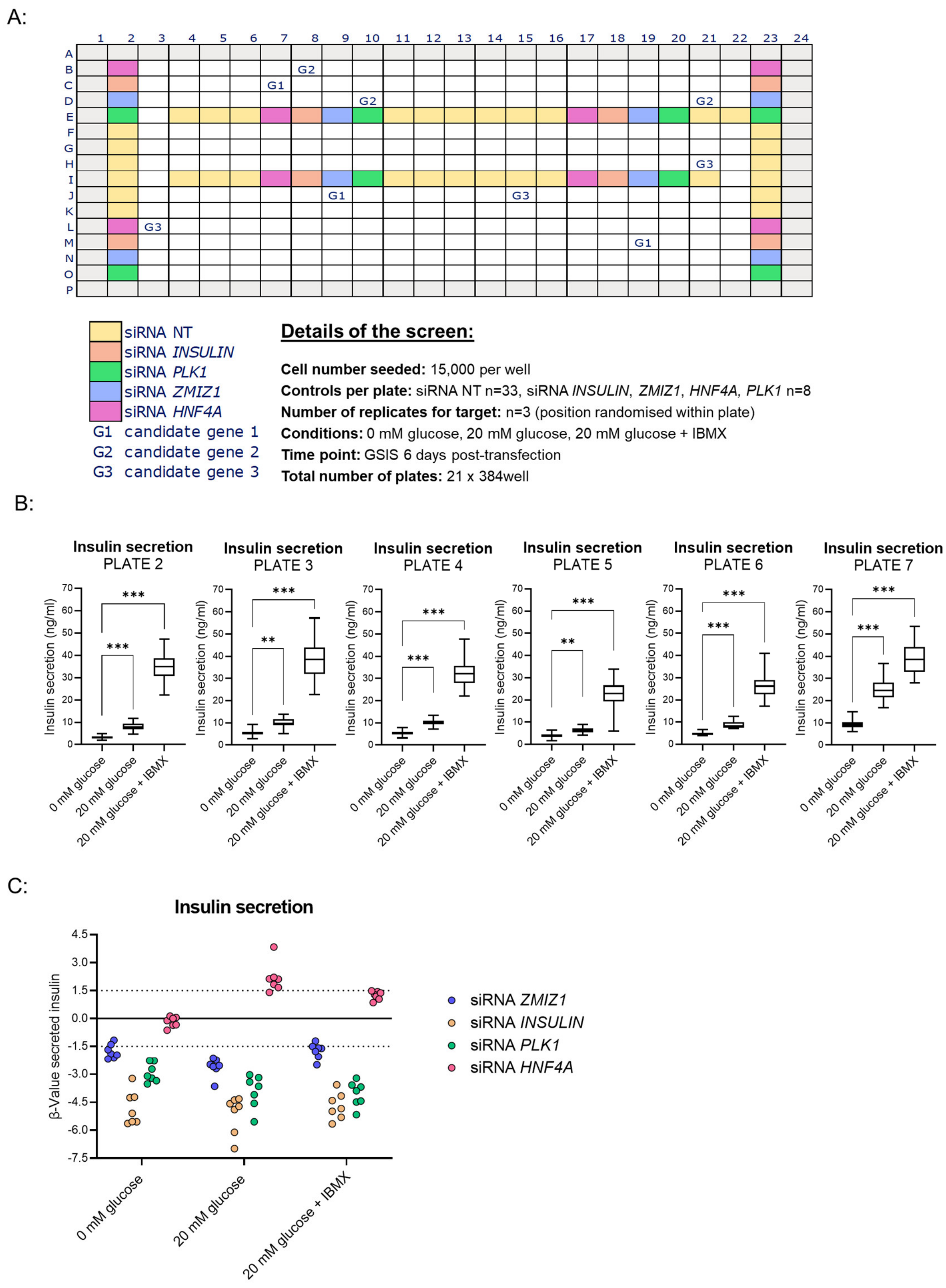

Figure S2: siRNA high-throughput screen in EndoC-βH1 cells with a GSIS readout**.** **(A)** Details of the siRNA high-throughput screen with a plate map example showing positions of controls (different colours) and randomized siRNAs positions for each replicate (n = 3) of text mining genes (candidate gene 1-3, G1-G3). **(B)** Insulin secretion data for non-targeting controls (siRNA NT) for Plates 2–7 in three conditions (0 mM glucose, 20 mM glucose, 20 mM glucose + IBMX). Box plots show min max of n = 33 for siNT. **p value <0.01, ***p value <0.001 by one-way ANOVA. **(C)** Dot plot of β–values for secreted insulin corresponding to controls on each plate. Each dot represents the β–value from a different assay plate (Plate 1 – Plate 7) in three conditions (0 mM glucose, 20 mM glucose, 20 mM glucose + IBMX).

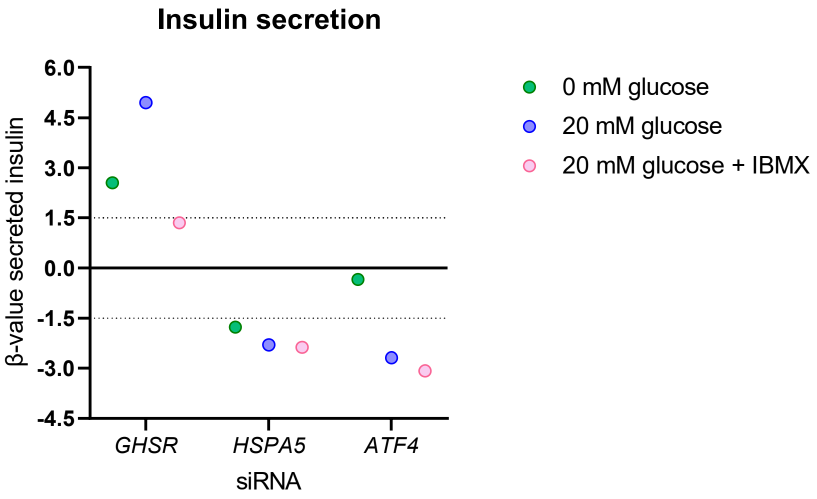

Figure S3. Insulin secretion (β–value) for the hits selected for validation. Dot plot depicting β–value for secreted insulin after knockdown of *GHSR, HSPA5* and *ATF4* in three GSIS conditions during the screen. Black dotted lines show selected β-value cut-offs of ≥1.5 and ≤−1.5.

Table S1. UniProt IDs of text mining genes screened in this study.

4EBP1_HUMAN	ADIPL_HUMAN	ANXA6_HUMAN	BDNF_HUMAN	CCKN_HUMAN
A4_HUMAN	ADIPO_HUMAN	AOFB_HUMAN	BECN1_HUMAN	CCL11_HUMAN
AA2AR_HUMAN	ADR1_HUMAN	APEL_HUMAN	BIP_HUMAN	CCL19_HUMAN
AAPK1_HUMAN	ADRB2_HUMAN	APLD1_HUMAN	BMAL1_HUMAN	CCL2_HUMAN
AAPK2_HUMAN	ADRB3_HUMAN	APOA_HUMAN	BMP4_HUMAN	CCL3_HUMAN
AATM_HUMAN	AGRB3_HUMAN	APOA1_HUMAN	BMP7_HUMAN	CCL4_HUMAN
ABCA1_HUMAN	AGRE1_HUMAN	APOA2_HUMAN	BSCL2_HUMAN	CCL5_HUMAN
ABCG1_HUMAN	AGRP_HUMAN	APOA4_HUMAN	C163A_HUMAN	CCN2_HUMAN
ABCG5_HUMAN	AGTR1_HUMAN	APOA5_HUMAN	C1QT1_HUMAN	CCN4_HUMAN
ABH15_HUMAN	AGTR2_HUMAN	APOB_HUMAN	C1QT3_HUMAN	CCND1_HUMAN
ABHD5_HUMAN	AKT1_HUMAN	APOC3_HUMAN	C1QT6_HUMAN	CCR2_HUMAN
ABHD6_HUMAN	AKT2_HUMAN	APOD_HUMAN	C1T9A_HUMAN	CCR5_HUMAN
ACACA_HUMAN	ALAT1_HUMAN	APOE_HUMAN	C2CD5_HUMAN	CD14_HUMAN
ACACB_HUMAN	AMYP_HUMAN	APOM_HUMAN	CAC1E_HUMAN	CD36_HUMAN
ACADL_HUMAN	AN36A_HUMAN	AQP7_HUMAN	CADM2_HUMAN	CD4_HUMAN
ACADM_HUMAN	ANDR_HUMAN	ARGI1_HUMAN	CART_HUMAN	CD40L_HUMAN
ACE_HUMAN	ANF_HUMAN	ARV1_HUMAN	CASP1_HUMAN	CD68_HUMAN
ACE2_HUMAN	ANFB_HUMAN	ATF3_HUMAN	CASP3_HUMAN	CEAM1_HUMAN
ACLY_HUMAN	ANGL3_HUMAN	ATF4_HUMAN	CATA_HUMAN	CEBPA_HUMAN
ACOD_HUMAN	ANGL4_HUMAN	ATF6A_HUMAN	CAV1_HUMAN	CEBPB_HUMAN
ACOX1_HUMAN	ANGL8_HUMAN	ATG5_HUMAN	CBPE_HUMAN	CERT_HUMAN
ACS2L_HUMAN	ANGT_HUMAN	ATG7_HUMAN	CBPM_HUMAN	CETP_HUMAN
ACTC_HUMAN	ANPRA_HUMAN	BCL2_HUMAN	CCD80_HUMAN	CFAD_HUMAN

CIDEA_HUMAN	DPP4_HUMAN	FTO_HUMAN	HGF_HUMAN	GTR9_HUMAN
CIDEC_HUMAN	DUPD1_HUMAN	G3P_HUMAN	HIF1A_HUMAN	GYS1_HUMAN
CISY_HUMAN	E2AK3_HUMAN	G6PC_HUMAN	HMDH_HUMAN	HDA11_HUMAN
CML1_HUMAN	EDN1_HUMAN	G6PD_HUMAN	HMGB1_HUMAN	HDAC7_HUMAN
CNR1_HUMAN	EF1A2_HUMAN	GALA_HUMAN	HMOX1_HUMAN	HEPC_HUMAN
CO1A1_HUMAN	EGF_HUMAN	GAST_HUMAN	HNF1A_HUMAN	HGF_HUMAN
CO3_HUMAN	EGFR_HUMAN	GCR_HUMAN	HNF1B_HUMAN	HIF1A_HUMAN
COG5_HUMAN	ELAF_HUMAN	GDF15_HUMAN	HPGDS_HUMAN	HMDH_HUMAN
COLI_HUMAN	ENC1_HUMAN	GDF8_HUMAN	HPT_HUMAN	HMGB1_HUMAN
COPA_HUMAN	ENHO_HUMAN	GHR_HUMAN	HS12A_HUMAN	HMOX1_HUMAN
COX41_HUMAN	EPO_HUMAN	GHRL_HUMAN	HSP74_HUMAN	HNF1A_HUMAN
CP19A_HUMAN	ERBB4_HUMAN	GHSR_HUMAN	HXK1_HUMAN	HNF1B_HUMAN
CP2E1_HUMAN	EREG_HUMAN	GIP_HUMAN	HXK2_HUMAN	HPGDS_HUMAN
CP7A1_HUMAN	ERF3B_HUMAN	GIPR_HUMAN	HXK3_HUMAN	HPT_HUMAN
CPT1A_HUMAN	ERN1_HUMAN	GLP1R_HUMAN	HXK4_HUMAN	HS12A_HUMAN
CPT1B_HUMAN	ESR1_HUMAN	GLR_HUMAN	IAPP_HUMAN	HSP74_HUMAN
CPT1C_HUMAN	F16P1_HUMAN	GLUC_HUMAN	IBP1_HUMAN	HXK1_HUMAN
CPT2_HUMAN	FABP4_HUMAN	GLUT4_HUMAN	IBP2_HUMAN	HXK2_HUMAN
CREG1_HUMAN	FABP5_HUMAN	GNA12_HUMAN	IBP3_HUMAN	HXK3_HUMAN
CRF_HUMAN	FABPH_HUMAN	GP119_HUMAN	ICAM1_HUMAN	HXK4_HUMAN
CRP_HUMAN	FABPI_HUMAN	GP142_HUMAN	IDE_HUMAN	IAPP_HUMAN
CRTC2_HUMAN	FABPL_HUMAN	GPBAR_HUMAN	IF2A_HUMAN	IBP1_HUMAN
CSF2_HUMAN	FAS_HUMAN	GPC5B_HUMAN	IF5A1_HUMAN	IBP2_HUMAN
CTNB1_HUMAN	FBX28_HUMAN	GPX1_HUMAN	IFNB_HUMAN	IBP3_HUMAN
CXB1_HUMAN	FETUA_HUMAN	GRB14_HUMAN	IFNG_HUMAN	ICAM1_HUMAN
CXCL2_HUMAN	FFAR1_HUMAN	GROA_HUMAN	IGF1_HUMAN	IDE_HUMAN
CXL10_HUMAN	FFAR4_HUMAN	GSHR_HUMAN	IGF1R_HUMAN	IF2A_HUMAN
CY24B_HUMAN	FGF1_HUMAN	GSK3A_HUMAN	IGF2_HUMAN	IF5A1_HUMAN
DCE1_HUMAN	FGF19_HUMAN	GSK3B_HUMAN	IKBA_HUMAN	IFNB_HUMAN
DCE2_HUMAN	FGF21_HUMAN	GSTP1_HUMAN	IKKA_HUMAN	IFNG_HUMAN
DDIT3_HUMAN	FGFP3_HUMAN	GTR1_HUMAN	IKKB_HUMAN	IGF1_HUMAN
DDX1_HUMAN	FGFR1_HUMAN	GTR10_HUMAN	IL10_HUMAN	IGF1R_HUMAN
DECR_HUMAN	FINC_HUMAN	GTR2_HUMAN	IL12A_HUMAN	IGF2_HUMAN
DGAT1_HUMAN	FITM2_HUMAN	GTR3_HUMAN	IL12B_HUMAN	IKBA_HUMAN
DGAT2_HUMAN	FKBP5_HUMAN	GTR4_HUMAN	IL13_HUMAN	IKKA_HUMAN
DGKE_HUMAN	FNDC5_HUMAN	GTR5_HUMAN	IL15_HUMAN	IKKB_HUMAN
DHI1_HUMAN	FOXA2_HUMAN	GTR9_HUMAN	IL17_HUMAN	IL10_HUMAN
DJC27_HUMAN	FOXA3_HUMAN	GYS1_HUMAN	IL18_HUMAN	IL12A_HUMAN
DLK1_HUMAN	FOXO1_HUMAN	HDA11_HUMAN	GTR3_HUMAN	IL12B_HUMAN
DNM1L_HUMAN	FOXO3_HUMAN	HDAC7_HUMAN	GTR4_HUMAN	IL13_HUMAN
DP13A_HUMAN	FOXP3_HUMAN	HEPC_HUMAN	GTR5_HUMAN	IL15_HUMAN

IL17_HUMAN	LIPL_HUMAN	NGAL_HUMAN	PER1_HUMAN	RETN_HUMAN
IL18_HUMAN	LIPP_HUMAN	NLRP3_HUMAN	PERM_HUMAN	RGS10_HUMAN
IL1A_HUMAN	LIPS_HUMAN	NOS2_HUMAN	PGBM_HUMAN	RHG21_HUMAN
IL1B_HUMAN	LOXE3_HUMAN	NOS3_HUMAN	PGH2_HUMAN	RHOA_HUMAN
IL1RA_HUMAN	LPIN1_HUMAN	NOTC1_HUMAN	PIN1_HUMAN	RICTR_HUMAN
IL2_HUMAN	LRP1_HUMAN	NOX1_HUMAN	PK3CA_HUMAN	RN186_HUMAN
IL2RA_HUMAN	LUM_HUMAN	NOX3_HUMAN	PK3CB_HUMAN	ROCK1_HUMAN
IL33_HUMAN	LYAM2_HUMAN	NOX4_HUMAN	PKNX1_HUMAN	RPGF3_HUMAN
IL37_HUMAN	LZTL1_HUMAN	NPY_HUMAN	PLCB_HUMAN	RPTOR_HUMAN
IL4_HUMAN	M3K7_HUMAN	NR0B2_HUMAN	PLGF_HUMAN	RS3A_HUMAN
IL6_HUMAN	MAGD1_HUMAN	NR1H3_HUMAN	PLIN1_HUMAN	RS6_HUMAN
IL7_HUMAN	MAGE1_HUMAN	NR1H4_HUMAN	PLIN2_HUMAN	RXRA_HUMAN
IL8_HUMAN	MARH1_HUMAN	NR1I2_HUMAN	PLIN5_HUMAN	S12A3_HUMAN
INSI1_HUMAN	MC4R_HUMAN	NRF1_HUMAN	PLOD1_HUMAN	S27A1_HUMAN
INSL5_HUMAN	MCR_HUMAN	NRG4_HUMAN	PLPL2_HUMAN	S39A5_HUMAN
INSR_HUMAN	METRL_HUMAN	NTCP2_HUMAN	PON1_HUMAN	SC5A1_HUMAN
IOD2_HUMAN	MGA_HUMAN	NUCB2_HUMAN	PPARA_HUMAN	SC5A2_HUMAN
IRS1_HUMAN	MGAT1_HUMAN	OCLN_HUMAN	PPARD_HUMAN	SCRB1_HUMAN
IRS2_HUMAN	MGLL_HUMAN	ODPX_HUMAN	PPARG_HUMAN	SEPT6_HUMAN
IRS4_HUMAN	MICU1_HUMAN	OGRL1_HUMAN	PRD16_HUMAN	SFRP5_HUMAN
ISL1_HUMAN	MK03_HUMAN	OLFM4_HUMAN	PRDM4_HUMAN	SHBG_HUMAN
ITAM_HUMAN	MK08_HUMAN	OREX_HUMAN	PRG4_HUMAN	SHIP2_HUMAN
ITAX_HUMAN	MKRN1_HUMAN	OSTCN_HUMAN	PRGC1_HUMAN	SIR1_HUMAN
ITLN1_HUMAN	MLXPL_HUMAN	OSTP_HUMAN	PRGC2_HUMAN	SIR3_HUMAN
JAK2_HUMAN	MMP2_HUMAN	OXYR_HUMAN	PRL_HUMAN	SIR6_HUMAN
JIP1_HUMAN	MMP9_HUMAN	P2RY2_HUMAN	PROS_HUMAN	SKIL_HUMAN
JUN_HUMAN	MOT11_HUMAN	P53_HUMAN	PTEN_HUMAN	SMAD2_HUMAN
K1C18_HUMAN	MRC1_HUMAN	PAFA_HUMAN	PTHY_HUMAN	SMAD3_HUMAN
KKCC2_HUMAN	MSRE_HUMAN	PAHO_HUMAN	PTN_HUMAN	SMS_HUMAN
KLOTB_HUMAN	MTOR_HUMAN	PAI1_HUMAN	PTN1_HUMAN	SNP23_HUMAN
KPCE_HUMAN	MTP_HUMAN	PAQR1_HUMAN	PUM3_HUMAN	SOAT1_HUMAN
KPYM_HUMAN	MYD88_HUMAN	PAQR2_HUMAN	PUR1_HUMAN	SOCS3_HUMAN
LAMA4_HUMAN	MYOD1_HUMAN	PARK7_HUMAN	PUR9_HUMAN	SODC_HUMAN
LBP_HUMAN	NAL12_HUMAN	PCKGC_HUMAN	PYC_HUMAN	SODM_HUMAN
LCAT_HUMAN	NALP2_HUMAN	PCNA_HUMAN	PYGO2_HUMAN	SPA12_HUMAN
LDLR_HUMAN	NAMPT_HUMAN	PCP_HUMAN	PYY_HUMAN	SRBP1_HUMAN
LEG3_HUMAN	NCF1_HUMAN	PCSK9_HUMAN	RAC1_HUMAN	SRBP2_HUMAN
LEP_HUMAN	NDUA5_HUMAN	PDE3B_HUMAN	RAGE_HUMAN	STA5A_HUMAN
LEPR_HUMAN	NEMF_HUMAN	PDK4_HUMAN	RARR2_HUMAN	STAT3_HUMAN
LFG3_HUMAN	NEU1_HUMAN	PDX1_HUMAN	RENI_HUMAN	STK11_HUMAN
LIPC_HUMAN	NF2L2_HUMAN	PEDF_HUMAN	RET4_HUMAN	SUCB1_HUMAN

SYVN1_HUMAN	TNFA_HUMAN	TXNIP_HUMAN	VASH1_HUMAN	XBP1_HUMAN
TBCD1_HUMAN	TNR1A_HUMAN	TY3H_HUMAN	VCAM1_HUMAN	XCR1_HUMAN
TBCD4_HUMAN	TNR5_HUMAN	UBC_HUMAN	VDR_HUMAN	XDH_HUMAN
TF7L2_HUMAN	TPC_HUMAN	UBP10_HUMAN	VEGFA_HUMAN	ZA2G_HUMAN
TFAM_HUMAN	TR11B_HUMAN	UBP19_HUMAN	VGFR2_HUMAN	ZHANG_HUMAN
TGFB1_HUMAN	TRH_HUMAN	UBP20_HUMAN	VLDLR_HUMAN	ZO1_HUMAN
TIMP1_HUMAN	TRIB3_HUMAN	UCP1_HUMAN	WDR13_HUMAN	
TLR2_HUMAN	TRPM2_HUMAN	UCP2_HUMAN	WNT5A_HUMAN	
TLR4_HUMAN	TSP1_HUMAN	UCP3_HUMAN	X3CL1_HUMAN	

Table S2. Catalog numbers of siRNAs used in this study.

Gene Name	ON-TARGETplus Human siRNA SMARTPool
INSULIN	L-011058-00-0005
ZMIZ1	L-007034-00-0005
HNF4A	L-003406-00-0005
PLK1	L-003290-00-0005
ATF4	L-005125-00-0005
HSPA5	L-008198-00-0005
GHSR	L-005513-00-0005

Table S3. Details of the Dharmacon master siRNA library. The Dharmacon master library plate sets contain the number of plates, wells and siRNAs as listed below. From these master plate sets, four sets of "daughter plates" are created containing a subset volume of 7.5 µL from the master plates in each well.

siRNA Master library	Number of plates	Number of wells	Number of siRNAs
Human Druggable Subsets Dharmacon, G-104675-E2	10	11,000	2750
Human Drug Targets Dharmacon, G-104655-E2	18	19,144	4786
Human Genome Dharmacon, G-106500-E2	38	41,680	10,420
Total	66	71,824	17,956

Table S4. Assay IDs of TaqMan probes used in the study.

Gene Name	Assay ID
TBP	Hs00427620_m1
INSULIN	Hs00355773_m1
ZMIZ1	Hs01119362_m1
HNF4A	Hs00230853_m1
ATF4	Hs00909569_g1
HSPA5	Hs00946087_g1
GHSR	Hs00269780_s1

Table S5: Hits from siRNA screen.

UniProt IDs	Gene Name	Plate Number	β Value Insulin Secretion 0 mM Glucose	β Value cell Number 0 mM Glucose	β Value Insulin Secretion 20 mM Glucose	β Value Cell Number 20 mM Glucose	β Value Insulin Secretion 20 mM Glucose + IBMX	β Value Cell Number 20 mM Glucose + IBMX
AA2AR_HUMAN	*ADORA2A*	1	−0.77	−0.40	−1.96	−0.46	−2.27	−0.33
ABHD6_HUMAN	*ABHD6*	5	−0.21	−0.58	2.01	−0.36	−0.02	−0.55
ADIPL_HUMAN	*FAM132A*	7	−1.88	−2.58	−1.37	−0.60	−1.70	−1.18
AGRP_HUMAN	*AGRP*	6	−1.74	−0.45	−0.06	−1.34	−0.36	−0.85
ANGL3_HUMAN	*ANGPTL3*	5	1.36	−0.24	2.94	−0.70	0.97	−0.22
ANGL4_HUMAN	*ANGPTL4*	7	−1.71	−0.24	−1.00	0.56	−1.53	−0.53
APOA5_HUMAN	*APOA5*	6	−1.43	0.48	−2.56	0.24	−0.39	−0.31
APOM_HUMAN	*APOM*	3	−1.40	−1.72	−0.30	−0.66	−1.77	−1.20
AQP7_HUMAN	*AQP7*	1	−0.72	0.08	−0.95	−0.67	−1.51	−0.40
ATF4_HUMAN	*ATF4*	4	−0.34	0.19	−2.69	−0.08	−3.08	−0.77
ATF6A_HUMAN	*ATF6*	4	0.12	0.54	1.72	0.58	−0.79	0.93
BIP_HUMAN	*HSPA5*	2	−1.77	0.19	−2.30	−0.57	−2.37	−0.70
CART_HUMAN	*CARTPT*	7	−1.70	−1.23	−1.43	−0.10	−1.76	−0.74
CASP3_HUMAN	*CASP3*	2	−0.78	0.68	−0.93	1.05	−1.71	−0.25
CCND1_HUMAN	*CCND1*	5	−1.25	−0.63	−1.82	−0.51	−0.72	−0.48
CCR5_HUMAN	*CCR5*	1	1.91	0.15	1.92	−0.14	1.06	0.02
CD14_HUMAN	*CD14*	4	3.97	0.08	1.56	0.31	1.74	0.10
CISY_HUMAN	*CS*	3	0.07	−0.28	−0.13	−0.65	−1.52	−0.69
CML1_HUMAN	*CMKLR1*	1	−0.08	−0.51	−0.48	0.22	−1.50	0.86
CREG1_HUMAN	*CREG1*	3	−0.85	−0.13	−0.08	0.14	−1.58	−0.30
CRTC2_HUMAN	*CRTC2*	6	−1.80	−0.70	−0.94	−0.81	−0.47	0.12
CSF2_HUMAN	*CSF2*	3	2.18	−1.89	1.05	−2.06	0.34	−2.67
CXL10_HUMAN	*CXCL10*	6	−1.67	−0.52	−0.62	−1.03	−0.98	−1.12
DGAT2_HUMAN	*DGAT2*	5	2.01	−1.62	0.48	−2.40	−0.88	−1.43
DUPD1_HUMAN	*DUPD1*	6	−1.70	−1.32	−2.58	−2.06	−1.26	−1.23
E2AK3_HUMAN	*EIF2AK3*	1	−0.70	1.34	−1.51	1.23	−0.05	1.23
ERBB4_HUMAN	*ERBB4*	1	0.02	0.76	1.56	−0.03	0.15	0.03
ERN1_HUMAN	*ERN1*	1	−1.51	−0.04	−1.93	−0.34	−3.04	−0.13
FETUA_HUMAN	*AHSG*	6	−0.31	−0.11	−1.59	−1.45	−1.24	−0.89
FGF19_HUMAN	*FGF19*	3	−0.71	−1.09	−0.48	−0.91	−1.52	−1.16
FGF21_HUMAN	*FGF21*	5	−0.10	0.48	−1.64	−0.03	−0.92	0.44
FOXA2_HUMAN	*FOXA2*	4	−3.45	−1.22	−3.50	−2.56	−3.43	−3.79
GHR_HUMAN	*GHR*	4	−1.48	−0.18	−1.34	0.13	−1.62	−0.58

GHRL_HUMAN	*GHRL*	6	−2.73	−1.17	−2.20	−3.08	−1.59	−1.61
GHSR_HUMAN	*GHSR*	1	2.55	−3.34	4.95	−3.47	1.35	−1.47
GLP1R_HUMAN	*GLP1R*	1	−1.31	−0.74	−2.06	−1.55	−3.13	−0.54
GPC5B_HUMAN	*GPRC5B*	1	−1.96	−1.40	−0.58	−2.22	−1.73	−2.05
GSK3B_HUMAN	*GSK3B*	1	1.55	2.39	0.76	0.67	−0.62	1.60
GTR5_HUMAN	*SLC2A5*	2	−0.87	1.52	−0.78	1.23	−1.55	0.57
GTR9_HUMAN	*SLC2A9*	2	1.38	−1.07	1.80	−1.12	0.58	−1.89
HEPC_HUMAN	*HAMP*	6	1.83	0.91	1.46	1.10	0.79	1.33
HGF_HUMAN	*HGF*	2	0.69	−0.73	2.60	−0.38	3.80	−0.20
HMDH_HUMAN	*HMGCR*	4	−1.71	−0.95	−1.27	−0.87	0.37	−0.57
HXK4_HUMAN	*GCK*	1	4.85	−0.24	0.30	−1.24	0.23	−0.24
IBP3_HUMAN	*IGFBP3*	6	−1.26	−0.35	−1.70	−1.36	−0.29	−0.40
IRS1_HUMAN	*IRS1*	5	−1.52	−0.55	−1.17	−0.76	−1.07	−0.29
JUN_HUMAN	*JUN*	3	−0.98	−0.91	−1.19	−0.48	−1.72	−0.89
KKCC2_HUMAN	*CAMKK2*	1	−0.08	−0.10	−0.55	0.76	−1.88	0.33
LOXE3_HUMAN	*ALOXE3*	2	−0.85	−1.50	−0.01	−1.58	−1.74	−1.01
MARH1_HUMAN	*MARC1*	2	−0.44	−0.73	−0.39	−0.54	−1.53	−0.41
MC4R_HUMAN	*MC4R*	1	−0.39	−1.08	−2.08	−1.71	−2.59	−0.71
MK03_HUMAN	*MAPK3*	1	0.68	−0.72	0.99	−1.79	3.12	−0.65
MMP2_HUMAN	*MMP2*	2	−1.71	0.42	−0.26	0.80	−0.49	−0.21
MMP9_HUMAN	*MMP9*	2	−1.17	0.79	−1.53	0.62	−1.26	−0.35
MSRE_HUMAN	*MSR1*	4	0.04	0.49	−1.00	−0.31	−1.99	−0.50
NCF1_HUMAN	*NCF1*	7	−1.17	−1.42	−2.87	−1.27	−2.95	−1.40
NEMF_HUMAN	*NEMF*	6	−1.47	−0.43	−1.63	−0.17	−1.82	−0.47
NF2L2_HUMAN	*NFE2L2*	3	−1.91	−1.00	−1.57	−1.04	−0.74	−2.22
NOS2_HUMAN	*NOS2*	3	−1.31	0.92	−0.33	0.89	−1.61	0.90
NR0B2_HUMAN	*NR0B2*	4	−0.90	−0.24	−1.73	0.19	−0.84	0.02
NRF1_HUMAN	*NRF1*	2	−1.23	−1.15	−1.24	−2.62	−2.36	−1.39
OXYR_HUMAN	*OXTR*	1	2.01	0.28	0.24	−0.89	0.67	0.15
PAQR2_HUMAN	*ADIPOR2*	6	−1.05	−0.52	−1.94	−1.02	−0.94	−0.35
PCSK9_HUMAN	*PCSK9*	2	2.14	0.21	1.03	−0.70	1.11	−0.18
PDE3B_HUMAN	*PDE3B*	4	−1.07	−1.29	−2.17	−1.99	−3.67	−1.28
PDK4_HUMAN	*PDK4*	1	1.79	−1.21	2.44	−0.97	−0.03	−0.42
PGH2_HUMAN	*PTGS2*	4	0.31	1.50	1.66	1.36	0.03	2.18
PK3CA_HUMAN	*PIK3CA*	1	−1.12	−0.38	−1.26	−1.52	−2.00	−0.25
PK3CB_HUMAN	*PIK3CB*	1	−1.14	−0.18	−1.46	−0.63	−1.67	−0.29
PLIN1_HUMAN	*PLIN1*	6	−0.42	−0.61	−1.69	−0.88	−0.50	−0.59
PON1_HUMAN	*PON1*	1	−1.62	−0.53	−1.44	−1.09	−0.72	−0.65
PRDM4_HUMAN	*PRDM4*	2	0.76	0.35	0.95	0.71	1.58	0.52
PRGC1_HUMAN	*PPARGC1A*	5	−0.83	−1.15	−1.53	−1.46	−1.40	−0.84
PUM3_HUMAN	*PUM3*	6	−1.27	−1.64	−1.65	−1.49	−0.60	−0.96

PYGO2_HUMAN	*PYGO2*	6	−1.46	−0.29	−1.64	−0.22	−0.52	−0.19
RPGF3_HUMAN	*RAPGEF3*	6	−1.27	−0.17	−0.48	−0.19	−1.69	−0.12
RPTOR_HUMAN	*RPTOR*	7	−1.44	0.08	−0.33	0.60	−1.50	−0.32
RXRA_HUMAN	*RXRA*	3	−0.53	−0.18	−0.51	0.31	−1.73	−0.55
SHBG_HUMAN	*SHBG*	6	0.58	−0.53	0.21	−0.88	−1.55	−0.90
SIR1_HUMAN	*SIRT1*	4	−0.90	0.36	−1.60	−0.03	−1.11	0.57
SOAT1_HUMAN	*SOAT1*	4	−0.85	−0.41	0.46	−0.69	−1.80	−0.62
SOCS3_HUMAN	*SOCS3*	2	2.23	−0.48	0.75	−0.96	2.05	0.00
SRBP2_HUMAN	*SREBF2*	3	−0.77	0.98	−1.65	1.32	−1.55	1.30
STA5A_HUMAN	*STAT5A*	3	−1.37	0.51	−1.63	1.10	−0.82	0.06
STK11_HUMAN	*STK11*	1	0.32	−0.16	1.79	−0.22	1.07	−0.14
TLR4_HUMAN	*TLR4*	4	−1.00	−0.79	−1.70	−0.45	0.07	−0.04
TR11B_HUMAN	*TNFRSF11B*	3	−0.15	−0.05	−0.61	−0.20	−1.82	−0.53
TRH_HUMAN	*TRH*	6	−0.76	0.30	−1.69	0.04	−0.41	0.04
TRIB3_HUMAN	*TRIB3*	1	−1.79	0.74	−1.51	−0.34	−1.20	−0.21
VASH1_HUMAN	*VASH1*	7	−1.82	−0.69	−0.41	−0.14	−1.38	−1.62
X3CL1_HUMAN	*CX3CL1*	7	0.39	0.71	−0.31	0.19	1.77	0.11
